# A multi locus variable number of tandem repeat analysis (MLVA) scheme for *Streptococcus agalactiae *genotyping

**DOI:** 10.1186/1471-2180-11-171

**Published:** 2011-07-27

**Authors:** Eve Haguenoer, Gaelle Baty, Christine Pourcel, Marie-Frédérique Lartigue, Anne-Sophie Domelier, Agnès Rosenau, Roland Quentin, Laurent Mereghetti, Philippe Lanotte

**Affiliations:** 1 Université François-Rabelais de Tours, UFR de Médecine, EA 3854 « Bactéries et risque materno-fœtal », Institut Fédératif de Recherche 136 « Agents Transmissibles et Infectiologie », Tours, France; 2 CHRU de Tours, Service de Bactériologie-Virologie, Tours, France; 3 Université Paris Sud 11, CNRS, UMR 8621, Institut de Génétique et Microbiologie, Orsay, 91405, France; 4 CHRU de Tours, Service de Bactériologie et d'Hygiène Hospitalière Tours, France

## Abstract

**Background:**

Multilocus sequence typing (MLST) is currently the reference method for genotyping *Streptococcus agalactiae *strains, the leading cause of infectious disease in newborns and a major cause of disease in immunocompromised children and adults. We describe here a genotyping method based on multiple locus variable number of tandem repeat (VNTR) analysis (MLVA) applied to a population of *S. agalactiae *strains of various origins characterized by MLST and serotyping.

**Results:**

We studied a collection of 186 strains isolated from humans and cattle and three reference strains (A909, NEM316 and 2603 V/R). Among 34 VNTRs, 6 polymorphic VNTRs loci were selected for use in genotyping of the bacterial population. The MLVA profile consists of a series of allele numbers, corresponding to the number of repeats at each VNTR locus. 98 MLVA genotypes were obtained compared to 51 sequences types generated by MLST. The MLVA scheme generated clusters which corresponded well to the main clonal complexes obtained by MLST. However it provided a higher discriminatory power. The diversity index obtained with MLVA was 0.960 compared to 0.881 with MLST for this population of strains.

**Conclusions:**

The MLVA scheme proposed here is a rapid, cheap and easy genotyping method generating results suitable for exchange and comparison between different laboratories and for the epidemiologic surveillance of *S. agalactiae *and analyses of outbreaks.

## Background

*Streptococcus agalactiae*, one of the group B streptococci (GBS), is a leading cause of bovine mastitis [1] and has been implicated in cases of invasive disease in humans since the 1960s and 1970s [2]. GBS have emerged as major pathogens in neonates [3] and in elderly adults, in whom they cause invasive infections, such as meningitis, soft tissue infections, endocarditis and osteoarticular infections [4,5]. There is a considerable body of evidence to suggest a genetic link between bovine isolates and the emerging human isolates [6,7].

GBS isolates were initially distinguished on the basis of differences in capsule polysaccharides, giving rise to 10 different serotypes [8,9]. Serotype III has been identified as a marker of late-onset neonatal disease isolates [10], but serotyping does not have sufficient discriminatory power to distinguish between isolates. Molecular methods have therefore been developed to determine the genetic relationships between isolates: multilocus enzyme electrophoresis [11], ribotyping [12], random amplified polymorphism DNA (RAPD) [13,14] and pulsed-field gel electrophoresis (PFGE) [15]. These methods make it possible to compare isolates and to define particular bacterial genogroups associated with invasive isolates in neonates. These findings were confirmed by multilocus sequence typing, as described by Jones *et al. *[16]. Other studies have shown that sequence type 17 (ST-17) isolates are associated with invasive behavior [17,18]. Two methods are currently used to explore the genetic links between isolates: PFGE for epidemiological studies, and MLST for both epidemiological and phylogenetic studies.

Analyses of fully sequenced bacterial genomes have revealed the existence of tandemly repeated sequences varying in size, location and the type of repetition [19]. Tandem repeats (TR) consist of a direct repetition of between one and more than 200 nucleotides, which may or may not be perfectly identical, located within or between genes. Depending on the size of the unit, the TR may be defined as a microsatellite (up to 9 bp) or a minisatellite (more than 9 bp) [19]. A fraction of these repeated sequences display intraspecies polymorphism and are described as VNTRs (variable number of tandem repeats). The proportion of VNTRs in the genome varies between bacterial species. Indeed, variation in the number of repeats at particular loci is used by some bacteria as a means of rapid genomic and phenotypic adaptation to the environment [20].

A molecular typing method based on VNTRs variability has recently been developed and applied to the typing of several bacterial pathogens [19]. Multiple locus VNTR analysis, or MLVA, is a PCR-based method that was originally developed for the typing of *Haemophilus influenzae *[21], *Mycobacterium tuberculosis *[22] and two bacterial species with potential for use in bioterrorism, *Bacillus anthracis *and *Yersinia pestis *[23,24]. This method has since been shown to be useful for the genotyping of several other bacterial species causing disease in humans, including *Streptococcus pneumoniae *[25], *Legionella pneumophila *[26], *Brucella *[27,28], *Pseudomonas aeruginosa *[29] and *Staphylococcus aureus *[30]. This technique has several advantages. For example, in bacterial species with high levels of genetic diversity, the study of six to eight markers is sufficient for accurate discrimination between strains [26]. Highly monomorphic species, such as *B. anthracis*, may be typed by MLVA, but this requires the use of a larger number of markers (25 VNTRs for *B. anthracis*) [31]. The discriminatory power of MLVA may also be increased by adding extra panels of more polymorphic markers [28] or by sequencing repeated sequences displaying internal variability [26]. Conversely, the evaluation of differences in the number of repeats only, on the basis of MLVA, is a cheap and rapid method that is not technically demanding. The work of Radtke *et al. *showed relevance of MLVA for *S. agalactiae *genotyping [32].

Our aim in this study was to develop a MLVA scheme for the genotyping of a population of *S. agalactiae *strains of various origins previously characterized by MLST.

## Methods

### Strains

Our collection consisted of 186 epidemiologically unrelated *S. agalactiae *strains, isolated from humans and cattle between 1966 and 2004 in France. Five of the 152 human strains were isolated from the gastric fluid of neonates, 71 were isolated from cases of vaginal carriage, 59 were isolated from cerebrospinal fluid and 17 were isolated from cultures of blood from adults presenting confirmed endocarditis according to the modified Duke criteria [33]. The 34 bovine strains were isolated from cattle presenting clinical signs of mastitis. We also studied three reference strains: NEM316, A909 and 2603 V/R. Each strain had previously been identified on the basis of Gram-staining, colony morphology, beta-hemolysis and Lancefield group antigen determination (Slidex Strepto Kit^®^, bioMérieux, Marcy l'Etoile, France). The capsular serotype was identified with the Pastorex^® ^rapid latex agglutination test (Bio-Rad, Hercules, USA) and by molecular serotyping, as described by Manning *et al. *[34]. We were unable to determine the serotype for 20 strains.

### DNA extraction

The bacteria were lysed mechanically with glass beads and their genomic DNA was extracted with an Invisorb^® ^Spin Cell Mini kit (Invitek, Berlin, Germany).

### MLST and assignment to clonal clusters

MLST was carried out as previously described [16]. Briefly, PCR was used to amplify small (≈ 500 bp) fragments from seven housekeeping genes (*adhP*, *pheS*, *atr*, *glnA*, *sdhA*, *glcK *and *tkt*) chosen on the basis of their chromosomal location and sequence diversity. The seven PCR products were purified and sequenced and an allele number was assigned to each fragment on the basis of its sequence. A sequence type (ST), based on the allelic profile of the seven amplicons, was assigned to each strain. The sequences of all new alleles and the composition of the new STs identified are available from http://pubmlst.org/sagalactiae/. Strains were grouped into clonal complexes (CCs) with eBURST software [35]. An eBURST clonal complex (CC) was defined as all allelic profiles sharing six identical alleles with at least one other member of the group. The term "singleton ST" refers to a ST that did not cluster into a CC.

### Identification of VNTR loci

Tandem repeats were identified in the sequenced genomes of the three reference strains, NEM316, A909 and 2603 V/R, with the Microbial Tandem Repeats Database http://minisatellites.u-psud.fr[36] and the Tandem Repeats Finder program [37]. We determined the size of the repeat sequence and the number of repeat units for the three reference strains. BLAST analysis was carried out to determine whether the repeats were located within or between genes and to identify a hypothetical function for the open reading frame involved. The TR locus name was defined according to the following nomenclature: common name_size of the repeat sequence_size of the amplicon for the reference strain_corresponding number of repeats (Table 1). The primers used for amplification targeted the 5' and 3' flanking regions of selected loci and matched the sequences present at these positions in the genomes of strains NEM316, A909 and 2603 V/R. We initially selected and evaluated 34 tandem repeats with repeat units of more than 9 bp in length. Some TRs were not present in all the strains, some were present in all strains and displayed no polymorphism, and others were too large for amplification in standard conditions. Six TRs were retained for this study, selected on the basis of their greater stability and discriminatory power for four of the six (Table 1).

**Table 1:** Characteristics of the 6 VNTR loci selected for MLVA scheme to genotype the 186 strains of *S. agalactiae*

**VNTR**^**1**^	**Repeat size bp**^**2**^	**Putative function**^**3**^	**Expected number of repeats**^**4**^			**PCR product bp**^**5**^	Number of alleles	min-max size of amplicons (bp)	***HGDI***^**6**^
			2603 V/R	A909	NEM316				
SAG2_32pb_244pb_3U	32	Non-cds^7^	3	3	3	244	3	212 - 276	0.474 [0.427 - 0.522]
SAG3_24pb_126pb_2U	24	Protein DnaJ	3	2	3	126	2	126 - 150	0.481 [0.452 - 0.511]
SAG4_60pb_114pb_1U (SATR1)*	60	Hypothetical protein	3	1	1	114	6	114 - 414	0.713 [0.691 - 0.735]
SAG7_18pb_285pb_8U (SATR2)*	18	Hypothetical protein	6	8	-	285	9	231-573	0.745 [0.701 - 0.789]
SAG21_48pb_783pb_14U (SATR5)*	48	FbsA	-	14	18	783	26	117 - ≈2000	0.893 [0.867 - 0.919]
SAG22_159pb_928pb_5U	159	Hypothetical protein	2	5	2	928	7	292 - 1246	0.713 [0.666 - 0.761]

^1^, Tandem repeat locus name defined as follows: common name_size of the repeat sequence_size of the amplicon for the A909 reference strain_corresponding number of repeats

^2^, Size of the repeat sequence

^3^, Putative function of the open reading frame concerned

^4^, 2603 V/R, A909 and NEM316 number of repeats (-: lack of VNTR)

^5^, Expected size of PCR product for the A909 reference strain

^6^, *HGDI*: Hunter and Gaston's diversity index, 95% confidence intervals are noted in brackets

7, 69 bp upstream from the ribosomal protein S10 sequence

*, Locus name described by Radtke

### Multiple locus VNTR analysis (MLVA)

The primers used for the VNTRs amplification are presented in Table 2. Three loci have already been described by Radtke *et al. *in a contemporary study but were amplified here with other primers [32] (Table 2). For the SAG7 locus, no amplification was observed with primers directly flanking the TR for 14% (26/189) of the strains. A second primer pair targeting larger consensual flanking regions was designed to confirm the absence of the locus. PCR was performed in a final volume of 25 μl containing 10 ng DNA, 1 × PCR Reaction Buffer, 2 mM MgCl_2 _(Applied Biosystems), 5% DMSO (dimethyl sulfoxide), 1 unit of *Taq *DNA polymerase (Applied Biosystems), 200 μM of each dNTP and 0.5 μM of each flanking primer (Eurogentec, Belgium). Amplification was performed in a 2720 Thermal Cycler (Applied Biosystems) under the following conditions: initial denaturation for 5 min at 94°C, followed by 30 cycles of denaturation for 30 s at 94°C, annealing for 30 s at 50°C and elongation for 60 s at 72°C plus a final elongation step for 7 min at 72°C. We separated 10 μl of PCR product by electrophoresis in a 2% agarose gel (Eurogentec, Belgium), which was also loaded with a 100 bp DNA size ladder (New England BioLabs). Electrophoresis was performed in 20 cm-long gels, in 1× TBE buffer (89 mM Tris-Borate, 2.5 mM EDTA) containing 1 μg/ml ethidium bromide run at 10 V/cm. In each run, at least one lane was loaded with PCR product from one of the reference strains, NEM316, A909 or 2603 V/R. The gels were photographed under ultraviolet illumination, with Vision-Capt^® ^Software (Vilber-Lourmat, Marne la Vallée, France). The number of repeats for each VNTR was deduced from amplicon size, by comparison with the reference strain, for which the number of repeats was known. The allele number corresponded to the number of repeats. For the SAG7 locus, the lack of a VNTR was revealed by the absence of amplification with the first primer pair and the amplification of a fragment of the expected size with the second primer pair, which targeted larger consensual flanking regions. In this case, an allele number of 0 was given. For the SAG21 locus, a 117 bp PCR product was obtained, demonstrating deletion of the inserted sequence and, thus, the absence of a VNTR. An allele number of 0 was also assigned in this case. The MLVA genotype of a strain was expressed as its allelic profile, corresponding to the number of repeats at each VNTR, listed in the order SAG2, SAG3, SAG4, SAG7, SAG21, SAG22.

**Table 2:** Primers used in the MLVA scheme

Forward primers	Sequence (5'-3')	Coordinates^1^	Ref strains^2^	Reverse primers	Sequence (5'-3')	Coordinates^1^
SAG2F	TCTTCCAAGTGGTGTCAACG	76270 - 76289	A909	SAG2R	CAACGTTTGGAGTTGCTTCA	76494 - 76513
SAG3F	CAAAAACGTGCTGCCTATGA	107351 - 107370	A909	SAG3R	CATCCCTCCTCCACCAAAA	107458 - 107476
SAG4F	GGTCAGTTTTTATTTATCGTAAGC	152991 - 153014	A909	SAG4R	AGTCTTGCGAAGGCAGACAC	153085 - 153104
SAG7F	TGGTGTTGATAAAGTTGATGTTCC	745963 - 745986	A909	SAG7R	GCCATATGAACTGCGGAAAC	746228 - 746247
SAG7bisF	ACCTATGCTCCCAGTGGTTC	111555 - 111574	NEM316	SAG7bisR	TCACTTAAGCGCACTGCAAC	112036 - 112055
SAG21F	TGAAAGAAGTGGATTTTTCCCTA	1062584 - 1062606	A909	SAG21R	AAAATAGGTTTTAGAACTTGGAAATCA	1062675 - 1062701
SAG22F	TGTAACACTAGCTCCAATTTGTTTT	1745819 - 1745843	A909	SAG22R	TCGGTCTTGTCTCAGCAATG	1746727 - 1746746

^1^, Nucleotide coordinates in the reference strains (A909, NEM316) chosen for primer design

^2^, Reference strain chosen for primer design

### Data analysis

The polymorphism index of individual or combined VNTR loci was calculated with the Hunter-Gaston diversity index [38], an application of Simpson's index of diversity [39]. Confidence intervals (CI) were calculated as described by Grundmann *et al. *[40]. The categorical coefficient (also called Hamming's distance) and unweighted pair group method with arithmetic mean (UPGMA) clustering approaches were run within BioNumerics. A cutoff value of 50% similarity was applied to define MLVA clusters. The minimum spanning tree (MST) was generated with BioNumerics. Each circle represents an MLVA genotype and its size is proportional to the number of strains. A logarithmic scale was used when drawing branches. The thicker branches link the MLVA genotypes differing by only one allele, the thinner branches link MLVA genotypes differing by more than one allele.

## Results

### MLST genotyping

MLST was performed on the 189 *S. agalactiae *strains, identifying a total of 51 individual STs. Eburst analysis clustered the STs into five clonal complexes (CC17, CC19, CC10, CC23 and CC7), two groups with only two STs and six singletons (Table 3). Two of the CCs -- CC17 (73 strains) and CC19 (63 strains) -- accounted for 72% (136/189) of the strains. CC23 accounted for 8% (15/189) of the strains. The various serotypes of *S. agalactiae *were distributed between multiple CCs and singleton STs. STs were characterized by a predominant serotype: serotype V in ST-1, serotype III in ST-17 and ST-19, serotype Ib in ST-10 and ST-12. ST-23 contained two serotypes (serotype Ia and III; Table 3). The population was therefore representative of *S. agalactiae *diversity in terms of anatomic origin, serotypes and clonal complexes (Table 3).

**Table 3:** Distribution of the 186 *S. agalactiae *strains studied and the 3 reference strains (NEM316, A909 and 2603 V/R), as a function of serotype and origin, within MLST clonal complexes

CC (No. of strains)	ST (No. of STs)	No. of strains (%)^1^	Serotype (No. of strains)	Origin of strains (No. of strains)					
				
				Vaginal carriage	Gastric fluid	Blood	Cerebro-spinal fluid	Bovine	Ref
**CC17 (73)**	17	56 (89%)	III (55), ND (1)	15	1	3	37	-	-
	Other (11)	17	II (5), III (6), ND (6)	2	-	-	1	14	-
	
**CC19 (63)**	19	27 (43%)	II (3), III (23), ND (1)	14	-	1	11	1	-
	1	12	V (11), ND (1)	10	-	1	1	-	-
	Other (13)	24	II (7), III (3), IV (4), V (3), ND (7)	11	3	3	1	5	1
									
	
**CC10 (17)**	10	9 (53%)	Ia (1), Ib (6), II (2)	5	-	2	1	1	-
	12	5	Ib (4), II (1)	3	-	2	-	-	-
	Other (2)	3	Ib (3)	1	-	1	1	-	-
	
**CC23 (15)**	23	10 (67%)	Ia (4), III (6)	2	-	1	4	2	1
	Other (5)	5	III (5)	1	-	2	-	2	-
	
**CC7 (9)**	7	5 (56%)	Ia (4), IV (1)	2	1	-	-	1	1
	Other (3)	4	Ib (3), V(1)	2	-	-	1	1	-
	
**ST-226**/**ST-314 (2)**		2	Ia (1), II (1)	1	-	-	-	1	-
**ST-300**/**ST-303 (2)**		2	ND (2)	-	-	-	-	2	-
**Singletons (8)**	Various (6)	8	II (2), III (2), V (2), ND (2)	2	-	1	1	4	-

**Total (189)**	51	189		71	5	17	59	34	3

**%^1^**, percentage of strains of the ST in the CC

ND: Not determinable

### Description of the MLVA scheme

**Figure 1:**
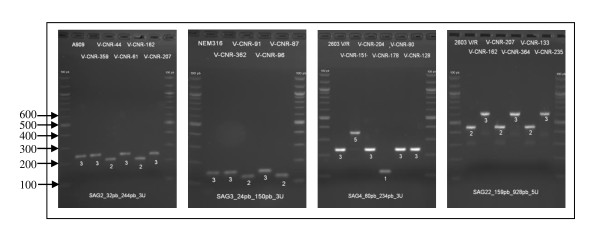
**Polymorphism of four VNTRs**. The polymorphism of VNTRs (SAG2, SAG3, SAG4 and SAG22) is shown by agarose gel electrophoresis of PCR products. The first strain on each gel is the reference strain and the PCR products were loaded alongside a 100 bp DNA size ladder (the sizes in base pairs are shown on the left side of the first panel). The allele number, corresponding to the number of repeats, is indicated under the band.

The six VNTRs were amplified from all 189 strains. MLVA was carried out with individual PCRs and agarose gel electrophoresis of the amplicons, as shown in Figure 1, for a subset of VNTRs. The repeat unit size of the six VNTRs was between 18 bp and 159 bp, making it straightforward to estimate the size of amplicons on agarose gels. For SAG2, SAG3, SAG4 and SAG7, amplicons were between 114 and 573 bp in size and were readily resolved by 2% agarose gel electrophoresis (Table 1). For SAG21 (48 bp repeat unit) and SAG22 (159 bp repeat unit), few amplicons exceeded 1,000 bp and extensive electrophoretic separation was required for precise estimations of size. For SAG21, three strains gave rise to amplicons of more than 1500 bp in size. This made it difficult to assess the number of repeats with any degree of precision, and an arbitrary allele number of > 30 was assigned in these cases. For SAG7, no amplification with the first primer pair was observed for 14% of strains. This locus is part of a genomic island and a second primer pair targeting consensual flanking regions beyond the borders of this genomic island was designed to confirm the deletion of the VNTR locus. The number of alleles was between two for SAG3 and 26 for SAG21. Thus, this MLVA method combined markers with a low discriminatory power (Hunter and Gaston's index of diversity or *HGDI *< 0.5) with highly discriminant markers, such as SAG21. With the exception of SAG2, the VNTRs used in this MLVA method were located within open reading frames (Table 1). SAG2 is located upstream from the gene encoding the ribosomal protein S10; SAG3 is located within *dnaJ*, encoding a co-chaperone protein (Hsp40). SAG21 is located within *fbsA*, encoding a protein involved in adhesion. SAG4, SAG7 and SAG22 are located in a "predicted coding region" of unknown function.

### MLVA genotyping and clustering

**Figure 2:**
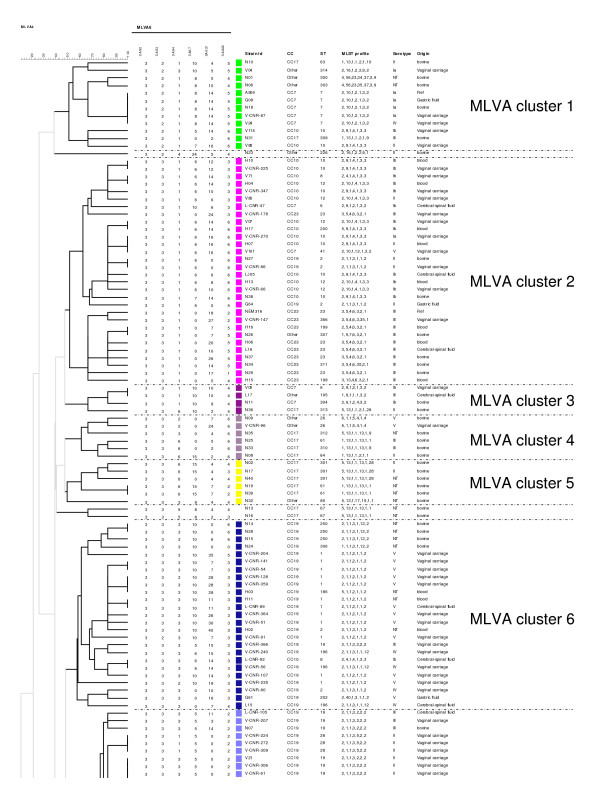
**MLVA clustering of the 189 strains of *S. agalactiae *by the UPGMA method, run in BioNumerics**. The names of strains (Strain Id), MLST clonal complex (CC), MLST sequence type (ST), MLST profile, serotype and the origin of strains are shown on the right. A cutoff value of 50% similarity was applied to define MLVA clusters (named MLVA cluster 1 to MLVA cluster 9). The colors used are based on MLVA clusters.

**Figure 3:**
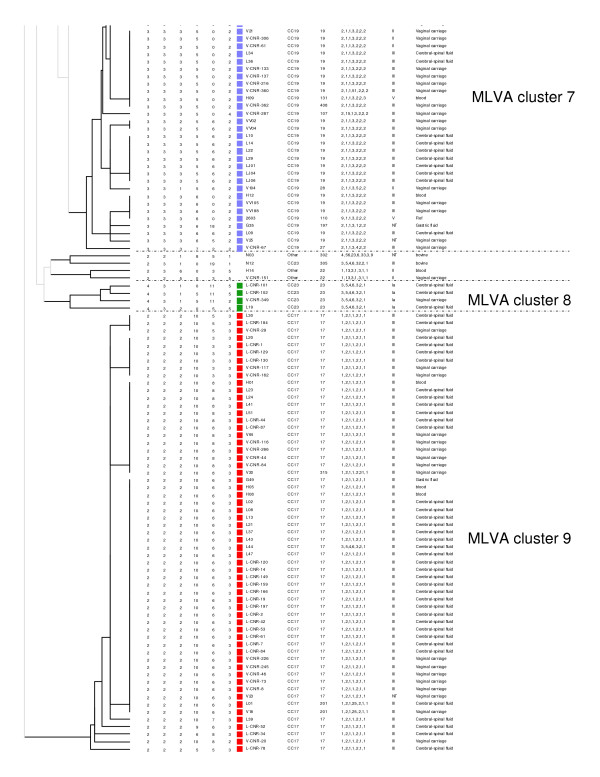
**MLVA clustering of the 189 strains of *S. agalactiae *by the UPGMA method, run in BioNumerics**. The names of strains (Strain Id), MLST clonal complex (CC), MLST sequence type (ST), MLST profile, serotype and the origin of strains are shown on the right. A cutoff value of 50% similarity was applied to define MLVA clusters (named MLVA cluster 1 to MLVA cluster 9). The colors used are based on MLVA clusters.

**Figure 4:**
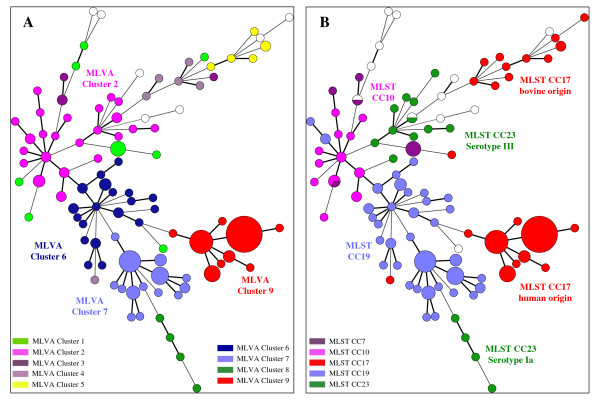
**Minimum spanning tree (MST) representation of the MLVA clustering**. The colors used in figure 4A are based on MLVA clusters. The colors used in figure 4B are based on MLST clonal complexes. White circles correspond to genotypes not clustered by MLVA or MLST. The MLVA data for 189 strains, including 3 reference strains, were analyzed in BioNumerics. Each circle represents an MLVA genotype and its size is proportional to the number of strains. A logarithmic scale was used when drawing branches. The thicker branches link the MLVA genotypes differing by only one allele, the thinner branches link MLVA genotypes differing by more than one allele.

The MLVA scheme resolved 98 genotypes among the 189 strains (Table 4). Five MLVA genotypes were represented by more than five strains: genotype 46 (n = 32), genotype 47 (n = 13), genotype 33 (n = 11), genotype 57 (n = 7) and genotype 51 (n = 6). Seventy-five MLVA genotypes were represented by only one strain (Table 4). *S. agalactiae *strains of different origins were spread among a number of MLVA genotypes. However 66% (39/59) of the strains isolated from cerebrospinal fluid were confined to four MLVA genotypes (genotypes 46, 47, 51 and 57). An MLVA cluster was defined by a cutoff value of 50% similarity with the UPGMA algorithm (Figures 2 and 3). Nine MLVA clusters, each containing more than four strains, were identified (MLVA clusters 1 to 9) (Figures 2 and 3 and Figure 4A). All clusters other than cluster 1 were congruent with the two algorithms, UPGMA and MST.

**Table 4:** MLVA genotypes resolved by the MLVA-6 scheme

MLVA Genotypes	Allelic Profile	Total (189)	Origin of strains (No. of strains)						STs (No. of strains)	Serotypes
					
			Vaginal carriage (71)	Gastric fluid (5)	Blood (17)	Cerebral-spinal fluid (59)	Bovine (34)	Ref (3)		
1	3,2,1,8,14,5	5	2	1	-	-	1	1	ST-7	Ia (4), IV
2	4,3,2,0,5,5	1	-	-	-	1	-	-	ST-23	Ia
3	4,3,1,0,11,5	1	-	-	-	1	-	-	ST-23	Ia
4	4,3,1,5,11,5	1	-	-	-	1	-	-	ST-23	Ia
5	3,2,3,10,5,5	1	1	-	-	-	-	-	ST-314	Ia
6	3,3,1,6,16,6	3	2	-	1	-	-	-	ST-10 (2), ST-41	Ia, II, V
7	4,3,1,5,11,2	1	1	-	-	-	-	-	ST-23	Ia
8	4,3,3,10,14,3	1	1	-	-	-	-	-	ST-1	V
9	3,3,1,10,9,4	1	-	-	-	-	1	-	ST-304	Ib
10	3,3,1,7,14,6	1	-	-	-	-	1	-	ST-10	Ib
11	3,3,1,6,12,3	2	1	-	1	-	-	-	ST-10	Ib
12	3,3,1,6,14,3	2	1	-	1	-	-	-	ST-8, ST-12	Ib
13	3,3,1,6,8,6	1	-	-	1	-	-	-	ST-12	Ib
14	3,3,1,6,14,6	2	1	-	1	-	-	-	ST-12, ST-200	Ib
15	3,3,1,10,6,3	1	-	-	-	1	-	-	ST-6	Ib
16	3,3,3,6,14,3	2	1	-	-	1	-	-	ST-8, ST-196	Ib, IV
17	3,3,1,6,6,6	1	-	-	-	1	-	-	ST-10	Ib
18	3,3,1,10,10,4	2	1	-	-	1	-	-	ST-6, ST-195	Ib, III
19	3,3,1,6,10,3	1	1	-	-	-	-	-	ST-10	Ib
20	3,3,1,6,10,6	1	1	-	-	-	-	-	ST-12	Ib
21	3,2,1,5,14,6	1	1	-	-	-	-	-	ST-10	Ib
22	3,3,6,15,4,3	1	-	-	-	-	1	-	ST-301	II
23	3,3,6,10,2,4	1	-	-	-	-	1	-	ST-313	II
24	3,3,6,15,4,4	1	-	-	-	-	1	-	ST-301	II
25	3,2,4,24,5,4	1	-	-	-	-	1	-	ST-226	II
26	3,2,1,10,4,5	1	-	-	-	-	1	-	ST-63	II
27	3,3,1,6,0,6	2	1	-	-	-	1	-	ST-2	II
28	3,3,6,15,2,6	1	-	-	-	-	1	-	ST-64	II
29	3,3,1,6,0,5	1	-	1	-	-	-	-	ST-2	II
30	2,3,6,0,3,5	1	-	-	1	-	-	-	ST-22	II
31	2,2,5,0,3,5	1	1	-	-	-	-	-	ST-22	II
32	3,3,1,5,0,2	3	3	-	-	-	-	-	ST-28	II
33	3,3,3,5,0,2	11	8	-	1	2	-	-	ST-19 (9), ST-131, ST-408	II (3), III (7), V
34	3,3,1,5,6,2	1	1	-	-	-	-	-	ST-28	II
35	3,3,1,6,6,3	1	1	-	-	-	-	-	ST-12	II
36	3,2,1,7,16,6	1	1	-	-	-	-	-	ST-10	II
37	2,3,1,0,19,1	1	-	-	-	-	1	-	ST-305	III
38	3,3,1,0,17,1	1	-	-	-	-	1	-	ST-23	III
39	3,3,3,5,14,2	1	-	-	-	-	1	-	ST-19	III
40	3,3,1,0,7,5	2	-	-	1	-	1	-	ST-199, ST-307	III
41	3,3,1,0,14,5	1	-	-	-	-	1	-	ST-311	III
42	3,3,1,0,26,5	1	-	-	-	-	1	-	ST-23	III
43	3,2,1,0,2,6	1	-	-	-	-	1	-	ST-309	III
44	3,3,6,0,2,6	1	-	-	-	-	1	-	ST-310	III
45	3,3,6,0,5,6	1	-	-	-	-	1	-	ST-61	III
46	2,2,2,10,6,3	32	7	1	2	22	-	-	ST-17 (30), ST-201	III (31), NT
47	2,2,2,10,8,3	13	6	-	1	6	-	-	ST-17 (12), ST-315	III
48	3,3,3,6,0,2	4	2	-	1	-	-	1	ST-19 (3), ST-110	III (3), V
49	3,3,1,0,5,4	1	-	-	1	-	-	-	ST-198	III
50	3,3,1,0,20,5	1	-	-	1	-	-	-	ST-23	III
51	2,2,2,10,3,3	6	2	-	-	4	-	-	ST-17	III
52	2,2,2,5,5,3	1	-	-	-	1	-	-	ST-17	III
53	2,2,2,10,5,3	3	1	-	-	2	-	-	ST-17	III
54	2,2,2,9,6,3	1	-	-	-	1	-	-	ST-17	III
55	2,2,2,10,7,3	1	-	-	-	1	-	-	ST-17	III
56	2,2,2,6,8,3	1	-	-	-	1	-	-	ST-17	III
57	3,3,3,5,6,2	7	-	-	-	7	-	-	ST-19	III
58	3,3,3,6,6,2	1	-	-	-	1	-	-	ST-19	III
59	3,3,3,5,11,2	1	-	-	-	1	-	-	ST-19	III
60	3,3,1,0,10,5	1	-	-	-	1	-	-	ST-23	III
61	3,3,1,0,18,2	1	-	-	-	-	-	1	ST-23	III
62	2,2,2,10,8,2	1	1	-	-	-	-	-	ST-17	III
63	3,3,3,7,2,2	1	1	-	-	-	-	-	ST-27	III
64	3,3,3,5,3,2	1	1	-	-	-	-	-	ST-19	III
65	3,3,2,5,6,2	2	2	-	-	-	-	-	ST-19	III
66	3,3,1,0,27,2	1	1	-	-	-	-	-	ST-366	III
67	3,3,3,10,7,3	2	2	-	-	-	-	-	ST-1	V
68	3,3,3,5,15,3	1	1	-	-	-	-	-	ST-19	III
69	3,3,1,0,24,3	1	1	-	-	-	-	-	ST-23	III
70	3,3,3,5,0,4	1	1	-	-	-	-	-	ST-107	III
71	3,3,3,0,7,4	1	-	-	-	1	-	-	ST-196	IV
72	3,3,3,0,10,3	1	1	-	-	-	-	-	ST-2	IV
73	3,3,3,6,15,3	1	1	-	-	-	-	-	ST-196	IV
74	2,2,1,8,5,1	1	-	-	-	-	1	-	ST-302	NT
75	3,3,6,15,7,2	2	-	-	-	-	2	-	ST-61	NT
76	3,3,5,8,2,3	1	-	-	-	-	1	-	ST-67	NT
77	3,3,6,0,4,4	1	-	-	-	-	1	-	ST-301	NT
78	3,3,5,8,4,4	1	-	-	-	-	1	-	ST-67	NT
79	3,3,3,9,4,4	1	-	-	-	-	1	-	ST-85	NT
80	3,2,1,8,5,4	1	-	-	-	-	1	-	ST-300	NT
81	3,2,1,8,10,4	1	-	-	-	-	1	-	ST-303	NT
82	3,3,3,10,0,6	2	-	-	-	-	2	-	ST-250	NT
83	3,3,3,0,4,6	1	-	-	-	-	1	-	ST-312	NT
84	3,3,3,10,6,6	1	-	-	-	-	1	-	ST-250	NT
85	3,3,3,10,0,7	1	-	-	-	-	1	-	ST-306	NT
86	3,3,3,6,18,2	1	-	1	-	-	-	-	ST-197	NT
87	3,3,3,10,11,3	2	-	-	1	1	-	-	ST-1	NT, V
88	3,3,3,10,28,3	3	2	-	1	-	-	-	ST-1, ST-186	NT, V
89	3,3,3,10,40,3	1	-	-	1	-	-	-	ST-2	NT
90	3,3,3,6,5,2	1	1	-	-	-	-	-	ST-19	NT
91	3,3,2,10,19,3	1	1	-	-	-	-	-	ST-1	V
92	3,3,2,0,7,6	1	-	-	-	-	1	-	ST-26	V
93	3,3,3,0,16,3	1	-	1	-	-	-	-	ST-202	V
94	3,2,3,10,7,3	1	1	-	-	-	-	-	ST-1	V
95	3,3,3,10,26,3	1	1	-	-	-	-	-	ST-1	V
96	3,3,3,10,30,3	1	1	-	-	-	-	-	ST-1	V
97	3,3,3,10,35,5	1	1	-	-	-	-	-	ST-1	V
98	3,3,2,0,24,6	1	1	-	-	-	-	-	ST-26	V

Each MLVA genotype is expressed as its allelic profile, corresponding to the number of repeats at each VNTR (SAG2, SAG3, SAG4, SAG7, SAG21 and SAG22)

### Comparison of MLVA and MLST clustering

MLVA clustering showed a clonal distribution of the population similar to that obtained by MLST (Figure 4). All human strains of MLST CC17 clustered together in MLVA cluster 9 and the bovine strains of MLST CC17 belonged to several MLVA clusters, suggesting greater heterogeneity of this population (Figure 4). With the exception of 3 strains, the MLST CC19 strains clustered into 2 linked MLVA clusters, MLVA cluster 6 and MLVA cluster 7. The MLST CC23 strains of serotype III and the MLST CC10 strains clustered into MLVA cluster 2. The strains from MLST CC23 serotype Ia also formed a separate group, the MLVA cluster 8.

### Discrimination of *S. agalactiae *strains by MLVA

The diversity index obtained with MLVA was 0.960 (95% CI [0.943 - 0.978]), which is greater than that obtained with MLST (0.881). For the population studied, MLVA distinguished 98 genotypes, whereas MLST distinguished 51 different STs. A much higher level of diversity was observed with MLVA, particularly within the major CCs. For example, the 73 CC17 strains were separated into 12 STs by MLST and 22 MLVA genotypes; the 63 CC19 strains were separated into 15 STs by MLST and 35 MLVA genotypes and the 15 CC23 strains were separated into 6 STs by MLST and 15 MLVA genotypes. Nevertheless, two genotypes (46 and 47) accounted for 76% (45/59) of CC17 strains of human origin. For this particular genogroup, the discriminatory power of the MLVA method was greater than that of MLST, although it remained low.

## Discussion

In this study, we applied the multi locus VNTR analysis (MLVA) typing method to *S. agalactiae*. VNTR analysis, a method based on tandem repeat polymorphisms at multiple loci, has been successfully applied to many other bacterial species [30,41]. We investigated the relevance of this tool for the genotyping of *S. agalactiae*, by testing this method on six VNTR loci in 189 strains previously characterized by MLST and serotyping. The MLVA-6 scheme is inexpensive and can be carried out with the equipment routinely used for PCR amplification and agarose gel electrophoresis. For the six VNTR loci, amplification was achieved with all the strains tested. For SAG7, a second PCR targeting a larger flanking region was required for 14% of the strains, which did not have a 16 kb genomic island encompassing the VNTR. The repeat sizes of the six VNTRs were sufficiently large for evaluation of the number of repeats on agarose gels. Moreover, the conversion of results into allelic profiles should make it possible to construct databases for exchange between laboratories. The MLVA-6 scheme includes a set of markers with different diversity indices, making it suitable for epidemiological studies. Markers with a moderate diversity and small number of alleles (presumably reflecting their slow rate of evolution) define clusters, whereas markers displaying more rapid evolution reflect variability within clusters. The MLVA-6 method described here is a rapid, reproducible and epidemiologically meaningful typing tool.

Three loci studied in the present MLVA scheme are in common with the MLVA scheme proposed by Radtke *et al. *[32]. The 3 additional loci studied here provide more weight to clusters while maintaining a high discrimination power. Moreover, in the MLVA scheme proposed here, only one locus (SAG7) was missing in some strains (14%), and another primer pair targeting larger consensual flanking region confirmed the absence of this locus with a specific amplification. Unlike Radtke *et al.*, we sought to develop a MLVA scheme in which a PCR product was amplified in all strains whether the VNTR was present or absent. In fact, negative amplification may result from the lack of a VNTR locus or modification of the flanking regions, especially as some VNTRs are close to transposases or insertion sequences such as SAG4 (alias SATR1) which is close to IS1381. Thus, the possibility of negative amplification for 3 out of 5 VNTR loci in the Radtke *et al. *MLVA analysis could be a real problem in terms of resolution and reproducibility of the genotyping method. Nevertheless, cumulative works allow to define the best set of VNTR loci, as has already been done for other bacterial species such as *Mycobacterium tuberculosis *[22,42-46] and *Staphylococcus aureus *[30,47-49]. Finally, the study of 34 isolates of bovine origin provided information about their distribution, especially those belonging to MLST CC17.

Population analysis by MLVA revealed a clonal distribution of the strains similar to that obtained by MLST. The greater discriminatory index of MLVA (0.96) made it possible to distinguish between strains within the clonal complexes defined by MLST. Thus, MLVA divided CC23 into two groups: one associated with serotype III and the other associated with serotype Ia. Moreover, MLVA also separated CC17 into two groups: one corresponding to strains of human origin and the other, containing several related STs (ST-61, ST-64, ST-301 etc.), corresponding to strains of animal origin only. A previous study analyzing 75 strains of *S. agalactiae *of human and animal origin by whole-genome DNA-array hybridization also separated ST-23 strains into two clusters, one associated with serotype III and the other with serotype Ia [50]. Each of these two clusters was associated with a particular pattern of surface protein expression. This previous study also separated the bovine and human CC17 strains [50]. These results are consistent with an ancient divergence of these clusters, whereas other observations based on MLST analysis suggest that ST-17 strains may have arisen from a bovine ancestor [6]. The lack of a strict correlation between the results of MLST and MLVA may be accounted for by differences in the markers used for MLST (targeting housekeeping genes) and MLVA (targeting a set of diverse regions that may or may not be conserved). Unlike MLST, MLVA targets several types of markers: genes involved in metabolism, genes associated with virulence and a genomic island. Indeed, SAG2 is located upstream from the gene encoding the ribosomal protein S10 which is involved in transcription and translation, and SAG3 is located within *dnaJ*, which encodes a member of the Hsp70 family, a co-chaperone protein (Hsp40). The SAG21 locus encodes a surface protein involved in virulence, FbsA. The SAG7 locus is located on a genomic island and belongs to a gene encoding a hypothetical protein whose function has not yet been identified, like most of the genes of genomic islands [51]. Clustering based on MLVA data was almost identical with the UPGMA and MST algorithms except for cluster 1. The differences in mathematical calculation between the two methods may account for the observed differences in strain clustering. This phenomenom has been previously observed in MLVA studies [52].

Some VNTRs for the alpha C protein have already been described in *S. agalactiae *[41,53,54]. One of these VNTRs is involved in regulating gene expression: a pentanucleotide repeat located upstream from the promoter regulates expression *in vitro *by phase variation. Another is an intragenic VNTR that modifies the size of the alpha C protein, thereby altering its antigenicity and strain virulence [53]. These two VNTR loci were not included in the MLVA method proposed here, in one case because the small size of the repeat unit (5 bp) complicates the mode of PCR fragment size assessment [19]. The amplicons of the second VNTR locus not included were more than 2000 bp in size, again making it difficult to evaluate repeat number. Tandem repeats were also found in the gene encoding another surface protein, FbsA, which interacts with epithelial cells and is involved in invasion of the central nervous system of colonized neonates. Its ability to bind to fibrinogen depends on the number of repeats of a unit of 16 amino acids present at its N-terminus [55]. A particular number of repeats is associated with the greater potential of the ST-17 strains implicated in neonatal meningitis to adhere to fibrinogen [56]. This major marker was included in our MLVA method and corresponds to SAG21.

## Conclusions

The MLVA method proposed here is a simple genotyping method producing results that can be exchanged between laboratories. MLVA generated major clusters that corresponded well to the main clonal complexes obtained by MLST. However its discriminatory power provided was greater that that of MLST. MLVA could also therefore be used as an epidemiological tool, given its high discriminatory power, making it possible to distinguish between strains of homogenous lineages. The specificities of the VNTRs for each phylogenetic lineage raise questions about the role of VNTRs in the adaptation of *S. agalactiae *to its environment and in virulence. Further studies are required to clarify these issues.

## Authors' contributions

EH and GB carried out the molecular genetic studies by MLST and MLVA. CP performed BioNumerics analysis of data and helped to draft the manuscript. MFL and ASD contributed to MLST analysis. AR and RQ participated in the design of the study. LM participated in the design of the study and helped to draft the manuscript. EH and PL conceived the study and draft the manuscript. All authors read and approved the final manuscript.

## References

[B1] Keefe GP *Streptococcus agalactiae *mastitis: a review Can Vet J 1997 38 429 437 9220132 PMC1576741

[B2] Schuchat A Group B streptococcal disease: from trials and tribulations to triumph and trepidation Clin Infect Dis 2001 33 751 756 10.1086/322697 11512078

[B3] Bohnsack JF Whiting A Gottschalk M Dunn DM Weiss R Azimi PH Philips JB Weisman LE Rhoads GG Lin F-YC Population structure of invasive and colonizing strains of *Streptococcus agalactiae *from neonates of six U.S. Academic Centers from 1995 to 1999 J Clin Microbiol 2008 46 1285 1291 10.1128/JCM.02105-07 18287314 PMC2292926

[B4] Edwards MS Rench MA Palazzi DL Baker CJ Group B streptococcal colonization and serotype-specific immunity in healthy elderly persons Clin Infect Dis 2005 40 352 357 10.1086/426820 15668856

[B5] Farley MM Group B streptococcal disease in nonpregnant adults Clin Infect Dis 2001 33 556 561 10.1086/322696 11462195

[B6] Bisharat N Crook DW Leigh J Harding RM Ward PN Coffey TJ Maiden MC Peto T Jones N Hyperinvasive neonatal group B streptococcus has arisen from a bovine ancestor J Clin Microbiol 2004 42 2161 2167 10.1128/JCM.42.5.2161-2167.2004 15131184 PMC404684

[B7] Héry-Arnaud G Bruant G Lanotte P Brun S Picard B Rosenau A van der Mee-Marquet N Rainard P Quentin R Mereghetti L Mobile genetic elements provide evidence for a bovine origin of clonal complex 17 of *Streptococcus agalactiae* Appl Environ Microbiol 2007 73 4668 4672 10.1128/AEM.02604-06 17526784 PMC1932819

[B8] Lindahl G Stålhammar-Carlemalm M Areschoug T Surface proteins of *Streptococcus agalactiae *and related proteins in other bacterial pathogens Clin Microbiol Rev 2005 18 102 127 10.1128/CMR.18.1.102-127.2005 15653821 PMC544178

[B9] Slotved H-C Kong F Lambertsen L Sauer S Gilbert GL Serotype IX, a proposed new *Streptococcus agalactiae *serotype J Clin Microbiol 2007 45 2929 2936 10.1128/JCM.00117-07 17634306 PMC2045254

[B10] Musser JM Mattingly SJ Quentin R Goudeau A Selander RK Identification of a high-virulence clone of type III *Streptococcus agalactiae *(group B Streptococcus) causing invasive neonatal disease Proc Natl Acad Sci USA 1989 86 4731 4735 10.1073/pnas.86.12.4731 2660146 PMC287347

[B11] Quentin R Huet H Wang FS Geslin P Goudeau A Selander RK Characterization of *Streptococcus agalactiae *strains by multilocus enzyme genotype and serotype: identification of multiple virulent clone families that cause invasive neonatal disease J Clin Microbiol 1995 33 2576 2581 8567885 10.1128/jcm.33.10.2576-2581.1995 PMC228531

[B12] Blumberg HM Stephens DS Licitra C Pigott N Facklam R Swaminathan B Wachsmuth IK Molecular epidemiology of group B streptococcal infections: use of restriction endonuclease analysis of chromosomal DNA and DNA restriction fragment length polymorphisms of ribosomal RNA genes (ribotyping) J Infect Dis 1992 166 574 579 10.1093/infdis/166.3.574 1380050

[B13] Chatellier S Huet H Kenzi S Rosenau A Geslin P Quentin R Genetic diversity of rRNA operons of unrelated *Streptococcus agalactiae *strains isolated from cerebrospinal fluid of neonates suffering from meningitis J Clin Microbiol 1996 34 2741 2747 8897176 10.1128/jcm.34.11.2741-2747.1996 PMC229397

[B14] Chatellier S Ramanantsoa C Harriau P Rolland K Rosenau A Quentin R Characterization of *Streptococcus agalactiae *strains by randomly amplified polymorphic DNA analysis J Clin Microbiol 1997 35 2573 2579 9316910 10.1128/jcm.35.10.2573-2579.1997 PMC230013

[B15] Rolland K Marois C Siquier V Cattier B Quentin R Genetic features of *Streptococcus agalactiae *strains causing severe neonatal infections, as revealed by pulsed-field gel electrophoresis and *hyl*B gene analysis J Clin Microbiol 1999 37 1892 1898 10325343 10.1128/jcm.37.6.1892-1898.1999 PMC84979

[B16] Jones N Bohnsack JF Takahashi S Oliver KA Chan M-S Kunst F Glaser P Rusniok C Crook DWM Harding RM Bisharat N Spratt BG Multilocus sequence typing system for group B streptococcus J Clin Microbiol 2003 41 2530 2536 10.1128/JCM.41.6.2530-2536.2003 12791877 PMC156480

[B17] Lamy M-C Dramsi S Billoët A Réglier-Poupet H Tazi A Raymond J Guérin F Couvé E Kunst F Glaser P Trieu-Cuot P Poyart C Rapid detection of the "highly virulent" group B Streptococcus ST-17 clone Microbes Infect 2006 8 1714 1722 10.1016/j.micinf.2006.02.008 16822689

[B18] Luan S-L Granlund M Sellin M Lagergård T Spratt BG Norgren M Multilocus sequence typing of Swedish invasive group B streptococcus isolates indicates a neonatally associated genetic lineage and capsule switching J Clin Microbiol 2005 43 3727 3733 10.1128/JCM.43.8.3727-3733.2005 16081902 PMC1233917

[B19] Lindstedt B-A Multiple-locus variable number tandem repeats analysis for genetic fingerprinting of pathogenic bacteria Electrophoresis 2005 26 2567 2582 10.1002/elps.200500096 15937984

[B20] Martin P van de Ven T Mouchel N Jeffries AC Hood DW Moxon ER Experimentally revised repertoire of putative contingency loci in *Neisseria meningitidis *strain MC58: evidence for a novel mechanism of phase variation Mol Microbiol 2003 50 245 257 10.1046/j.1365-2958.2003.03678.x 14507378

[B21] Van Belkum A Melchers WJ Ijsseldijk C Nohlmans L Verbrugh H Meis JF Outbreak of amoxicillin-resistant *Haemophilus influenzae *type b: variable number of tandem repeats as novel molecular markers J Clin Microbiol 1997 35 1517 1520 9163472 10.1128/jcm.35.6.1517-1520.1997 PMC229777

[B22] Supply P Mazars E Lesjean S Vincent V Gicquel B Locht C Variable human minisatellite-like regions in the *Mycobacterium tuberculosis *genome Mol Microbiol 2000 36 762 771 10844663 10.1046/j.1365-2958.2000.01905.x

[B23] Keim P Price LB Klevytska AM Smith KL Schupp JM Okinaka R Jackson PJ Hugh-Jones ME Multiple-locus variable-number tandem repeat analysis reveals genetic relationships within *Bacillus anthracis* J Bacteriol 2000 182 2928 2936 10.1128/JB.182.10.2928-2936.2000 10781564 PMC102004

[B24] Le Flèche P Hauck Y Onteniente L Prieur A Denoeud F Ramisse V Sylvestre P Benson G Ramisse F Vergnaud G A tandem repeats database for bacterial genomes: application to the genotyping of *Yersinia pestis *and *Bacillus anthracis* BMC Microbiol 2001 1 2 10.1186/1471-2180-1-2 11299044 PMC31411

[B25] Koeck J-L Njanpop-Lafourcade B-M Cade S Varon E Sangare L Valjevac S Vergnaud G Pourcel C Evaluation and selection of tandem repeat loci for *Streptococcus pneumoniae *MLVA strain typing BMC Microbiol 2005 5 66 10.1186/1471-2180-5-66 16287512 PMC1315331

[B26] Pourcel C Visca P Afshar B D'Arezzo S Vergnaud G Fry NK Identification of variable-number tandem-repeat (VNTR) sequences in *Legionella pneumophila *and development of an optimized multiple-locus VNTR analysis typing scheme J Clin Microbiol 2007 45 1190 1199 10.1128/JCM.02078-06 17251393 PMC1865833

[B27] Al Dahouk S Flèche PL Nöckler K Jacques I Grayon M Scholz HC Tomaso H Vergnaud G Neubauer H Evaluation of *Brucella *MLVA typing for human brucellosis J Microbiol Methods 2007 69 137 145 10.1016/j.mimet.2006.12.015 17261338

[B28] Le Flèche P Jacques I Grayon M Al Dahouk S Bouchon P Denoeud F Nöckler K Neubauer H Guilloteau LA Vergnaud G Evaluation and selection of tandem repeat loci for a *Brucella *MLVA typing assay BMC Microbiol 2006 6 1471 1484 10.1186/1471-2180-6-9 PMC1513380 16469109

[B29] Vu-Thien H Corbineau G Hormigos K Fauroux B Corvol H Clément A Vergnaud G Pourcel C Multiple-locus variable-number tandem-repeat analysis for longitudinal survey of sources of *Pseudomonas aeruginosa *infection in cystic fibrosis patients J Clin Microbiol 2007 45 3175 3183 10.1128/JCM.00702-07 17699654 PMC2045346

[B30] Pourcel C Hormigos K Onteniente L Sakwinska O Deurenberg RH Vergnaud G Improved multiple-locus variable-number tandem-repeat assay for *Staphylococcus aureus *genotyping, providing a highly informative technique together with strong phylogenetic value J Clin Microbiol 2009 47 3121 3128 10.1128/JCM.00267-09 19710277 PMC2756900

[B31] Lista F Faggioni G Valjevac S Ciammaruconi A Vaissaire J le Doujet C Gorgé O De Santis R Carattoli A Ciervo A Fasanella A Orsini F D'Amelio R Pourcel C Cassone A Vergnaud G Genotyping of *Bacillus anthracis *strains based on automated capillary 25-loci multiple locus variable-number tandem repeats analysis BMC Microbiol 2006 6 33 10.1186/1471-2180-6-33 16600037 PMC1479350

[B32] Radtke A Lindstedt B-A Afset JE Bergh K Rapid multiple-locus variant-repeat assay (MLVA) for genotyping of *Streptococcus agalactiae* J Clin Microbiol 2010 48 2502 2508 10.1128/JCM.00234-10 20504982 PMC2897478

[B33] Li JS Sexton DJ Mick N Nettles R Fowler VG Ryan T Bashore T Corey GR Proposed modifications to the Duke criteria for the diagnosis of infective endocarditis Clin Infect Dis 2000 30 633 638 10.1086/313753 10770721

[B34] Manning SD Lacher DW Davies HD Foxman B Whittam TS DNA polymorphism and molecular subtyping of the capsular gene cluster of group B streptococcus J Clin Microbiol 2005 43 6113 6116 10.1128/JCM.43.12.6113-6116.2005 16333106 PMC1317180

[B35] Feil EJ Li BC Aanensen DM Hanage WP Spratt BG eBURST: inferring patterns of evolutionary descent among clusters of related bacterial genotypes from multilocus sequence typing data J Bacteriol 2004 186 1518 1530 10.1128/JB.186.5.1518-1530.2004 14973027 PMC344416

[B36] Denoeud F Vergnaud G Identification of polymorphic tandem repeats by direct comparison of genome sequence from different bacterial strains: a web-based resource BMC Bioinformatics 2004 5 4 10.1186/1471-2105-5-4 14715089 PMC331396

[B37] Benson G Tandem repeats finder: a program to analyze DNA sequences Nucleic Acids Res 1999 27 573 580 10.1093/nar/27.2.573 9862982 PMC148217

[B38] Hunter PR Gaston MA Numerical index of the discriminatory ability of typing systems: an application of Simpson's index of diversity J Clin Microbiol 1988 26 2465 2466 3069867 10.1128/jcm.26.11.2465-2466.1988 PMC266921

[B39] Simpson EH Measurement of diversity Nature 1949 163 688 10.1038/163688a0

[B40] Grundmann H Hori S Tanner G Determining confidence intervals when measuring genetic diversity and the discriminatory abilities of typing methods for microorganisms J Clin Microbiol 2001 39 4190 4192 10.1128/JCM.39.11.4190-4192.2001 11682558 PMC88515

[B41] Puopolo KM Madoff LC Upstream short sequence repeats regulate expression of the alpha C protein of group B Streptococcus Mol Microbiol 2003 50 977 991 10.1046/j.1365-2958.2003.03745.x 14617155

[B42] Frothingham R Meeker-O'Connell WA Genetic diversity in the *Mycobacterium tuberculosis *complex based on variable numbers of tandem DNA repeats Microbiology 1998 144 1189 1196 10.1099/00221287-144-5-1189 9611793

[B43] Supply P Lesjean S Savine E Kremer K van Soolingen D Locht C Automated high-throughput genotyping for study of global epidemiology of *Mycobacterium tuberculosis *based on mycobacterial interspersed repetitive units J Clin Microbiol 2001 39 3563 3571 10.1128/JCM.39.10.3563-3571.2001 11574573 PMC88389

[B44] Mazars E Lesjean S Banuls AL Gilbert M Vincent V Gicquel B Tibayrenc M Locht C Supply P High-resolution minisatellite-based typing as a portable approach to global analysis of *Mycobacterium tuberculosis *molecular epidemiology Proc Natl Acad Sci USA 2001 98 1901 1906 10.1073/pnas.98.4.1901 11172048 PMC29354

[B45] Le Flèche P Fabre M Denoeud F Koeck J-L Vergnaud G High resolution, on-line identification of strains from the *Mycobacterium tuberculosis *complex based on tandem repeat typing BMC Microbiol 2002 2 37 10.1186/1471-2180-2-37 12456266 PMC140014

[B46] Supply P Allix C Lesjean S Cardoso-Oelemann M Rüsch-Gerdes S Willery E Savine E de Haas P van Deutekom H Roring S Bifani P Kurepina N Kreiswirth B Sola C Rastogi N Vatin V Gutierrez MC Fauville M Niemann S Skuce R Kremer K Locht C van Soolingen D Proposal for standardization of optimized mycobacterial interspersed repetitive unit-variable-number tandem repeat typing of *Mycobacterium tuberculosis* J Clin Microbiol 2006 44 4498 4510 10.1128/JCM.01392-06 17005759 PMC1698431

[B47] Sabat A Krzyszton-Russjan J Strzalka W Filipek R Kosowska K Hryniewicz W Travis J Potempa J New method for typing *Staphylococcus aureus *strains: multiple-locus variable-number tandem repeat analysis of polymorphism and genetic relationships of clinical isolates J Clin Microbiol 2003 41 1801 1804 10.1128/JCM.41.4.1801-1804.2003 12682193 PMC153872

[B48] Francois P Huyghe A Charbonnier Y Bento M Herzig S Topolski I Fleury B Lew D Vaudaux P Harbarth S van Leeuwen W van Belkum A Blanc DS Pittet D Schrenzel J Use of an automated multiple-locus, variable-number tandem repeat-based method for rapid and high-throughput genotyping of *Staphylococcus aureus *isolates J Clin Microbiol 2005 43 3346 3355 10.1128/JCM.43.7.3346-3355.2005 16000459 PMC1169139

[B49] Hardy KJ Ussery DW Oppenheim BA Hawkey PM Distribution and characterization of staphylococcal interspersed repeat units (SIRUs) and potential use for strain differentiation Microbiology 2004 150 4045 4052 10.1099/mic.0.27413-0 15583157

[B50] Brochet M Couvé E Zouine M Vallaeys T Rusniok C Lamy M-C Buchrieser C Trieu-Cuot P Kunst F Poyart C Glaser P Genomic diversity and evolution within the species *Streptococcus agalactiae* Microbes Infect 2006 8 1227 1243 10.1016/j.micinf.2005.11.010 16529966

[B51] Tettelin H Genome analysis of multiple pathogenic isolates of *Streptococcus agalactiae*: implications for the microbial "pan-genome" Proc Natl Acad Sci USA 2005 102 13950 13955 10.1073/pnas.0506758102 16172379 PMC1216834

[B52] Dauchy FA Degrange S Charron A Dupon M Xin Y Bebear C Maugein J Variable-number tandem-repeat markers for typing *Mycobacterium intracellulare *strains isolated in humans BMC Microbiol 2010 10 93 10.1186/1471-2180-10-93 20350295 PMC2861668

[B53] Gravekamp C Kasper DL Michel JL Kling DE Carey V Madoff LC Immunogenicity and protective efficacy of the alpha C protein of group B streptococci are inversely related to the number of repeats Infect Immun 1997 65 5216 5221 9393818 10.1128/iai.65.12.5216-5221.1997 PMC175751

[B54] Madoff LC Michel JL Gong EW Kling DE Kasper DL Group B streptococci escape host immunity by deletion of tandem repeat elements of the alpha C protein Proc Natl Acad Sci USA 1996 93 4131 4136 10.1073/pnas.93.9.4131 8633028 PMC39499

[B55] Schubert A Zakikhany K Schreiner M Frank R Spellerberg B Eikmanns BJ Reinscheid DJ A fibrinogen receptor from group B Streptococcus interacts with fibrinogen by repetitive units with novel ligand binding sites Mol Microbiol 2002 46 557 569 10.1046/j.1365-2958.2002.03177.x 12406229

[B56] Rosenau A Martins K Amor S Gannier F Lanotte P van der Mee-Marquet N Mereghetti L Quentin R Evaluation of the ability of *Streptococcus agalactiae *strains isolated from genital and neonatal specimens to bind to human fibrinogen and correlation with characteristics of the *fbsA *and *fbsB *genes Infect Immun 2007 75 1310 1317 10.1128/IAI.00996-06 17158903 PMC1828567

